# Supplementary material for: Evidence supported by Mendelian randomization: impact on inflammatory factors in knee osteoarthritis
Source: Front Med (Lausanne). 2024 May 28;11:1382836. doi: 10.3389/fmed.2024.1382836 (PMC11165061; doi:10.3389/fmed.2024.1382836)
Supplement: Supplementary file 1 [file Data_Sheet_1.ZIP › data/GCST90274797_5e6/797.forest.pdf]

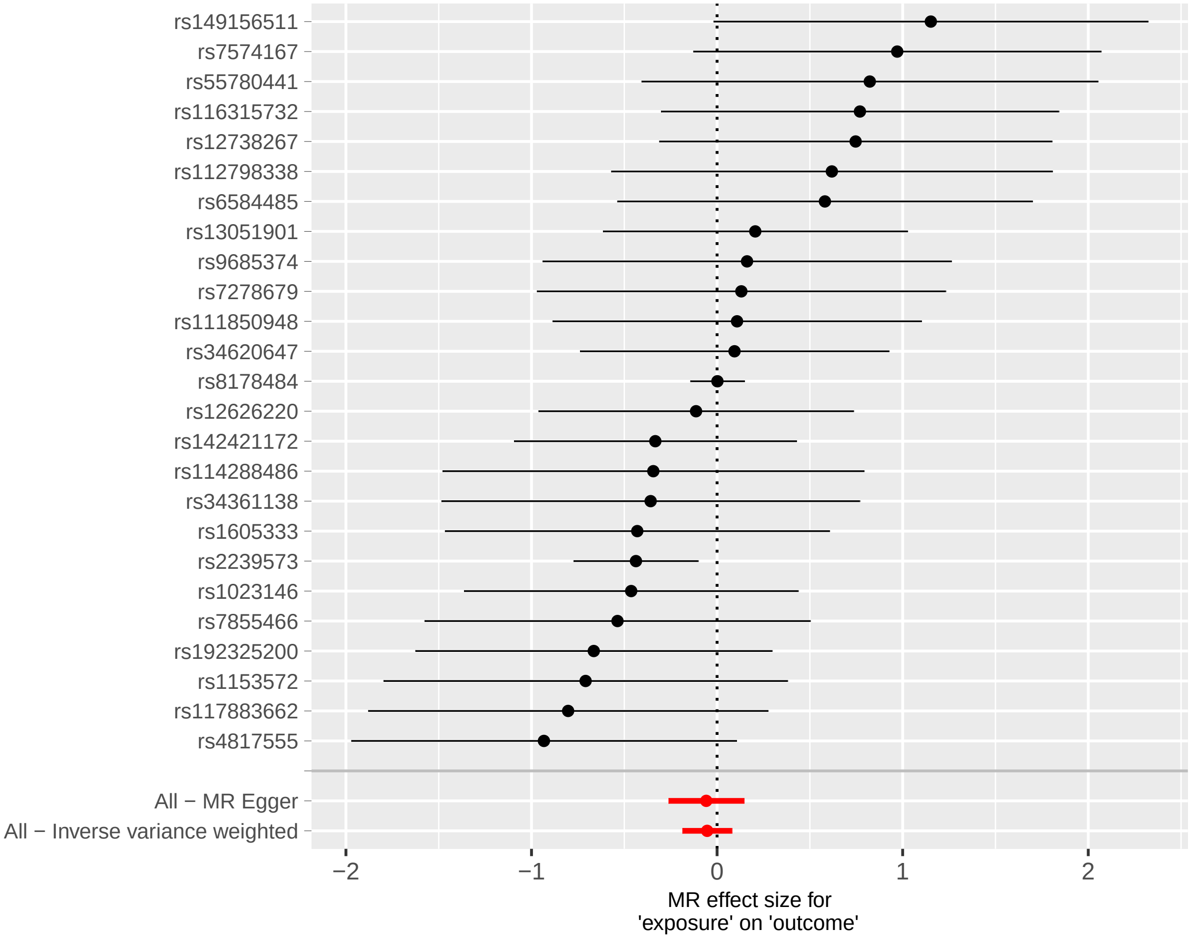
rs149156511
rs7574167
rs55780441
rs116315732
rs12738267
rs112798338
rs6584485
rs13051901
rs9685374
rs7278679
rs111850948
rs34620647
rs8178484
rs12626220
rs142421172
rs114288486
rs34361138
rs1605333
rs2239573
rs1023146
rs7855466
rs192325200
rs1153572
rs117883662
rs4817555
All − MR Egger
All − Inverse variance weighted
−2
−1
0
1
2
MR effect size for
'exposure' on 'outcome'
